# Pediatric Congenital Lung Malformation: Advanced Imaging Techniques in Pre- and Neonatal Evaluation

**DOI:** 10.3390/diagnostics15091112

**Published:** 2025-04-27

**Authors:** Gabriele Masselli, Chiara Di Bella, George Hadjidekov, Carlos Carnelli, Francesco Morini, Silvia Ceccanti, Fabio Midulla, Denis A. Cozzi

**Affiliations:** 1 Department of Radiological Sciences, Oncology and Pathology, Policlinico Umberto I, Sapienza University of Rome, 00161 Rome, Italy; chiaradibella30@gmail.com; 2 Department of Physics, Biophysics and Radiology, Medical Faculty, Sofia University St. Kliment Ohridski, 1407 Sofia, Bulgaria; jordiman76@yahoo.com; 3 Hospital de Clínicas, Universidad de la República, Montevideo 11200, Uruguay; carnelli.carlos@gmail.com; 4 Pediatric Surgery Unit, Sapienza University of Rome, Azienda Ospedaliero-Universitaria Policlinico Umberto I, 00161 Rome, Italy; francesco.morini@uniroma1.it (F.M.); silvia.ceccanti@uniroma1.it (S.C.); da.cozzi@uniroma1.it (D.A.C.); 5 Department of Maternal Science and Urology, Sapienza University of Rome, 00185 Rome, Italy; fabio.midulla@uniroma1.it

**Keywords:** congenital malformation, lung, imaging, CT, MRI, children, fetal, pediatric

## Abstract

Pediatric congenital lung malformations (CLMs) comprise a spectrum of developmental anomalies of lung parenchyma, airways, and vasculature. CLMs are increasingly diagnosed prenatally but remain best characterized by postnatal cross-sectional imaging. During pregnancy, ultrasound (US) and fetal magnetic resonance imaging (MRI) are commonly used to monitor lung lesions. Management of CLMs, including imaging, in infants and young children depends on associated symptoms and institutional standards. Chest CT angiography (CTA) is usually the most appropriate initial postnatal imaging modality for assessing prenatally diagnosed or clinically suspected CLMs in asymptomatic infants and children. Magnetic resonance (MR) imaging/magnetic resonance angiography (MRA) may be considered as a complementary, problem-solving, imaging modality for evaluation of CLMs during fetal and neonatal periods. This article presents contemporary perspectives on the imaging approach to pediatric patients with suspected CLMs and reviews up-to-date radiologic findings and clinical characteristics of CLMs.

## 1. Introduction

Congenital lung malformations (CLMs) refer to a variety of developmental disorders that affect the lung tissue, airways, and blood vessels [1]. These malformations are being identified more frequently, particularly during pregnancy, with an incidence now estimated at up to 4 in 10,000 live births [2].

Many CLMs are considered part of a broader spectrum that involves in utero airway obstruction and lung malformation, often accompanied by vascular anomalies [3]. A thorough evaluation of all components—airway, lung tissue, and vasculature—is essential for accurately identifying, classifying, and managing these lesions. Such assessments are crucial for making informed decisions regarding diagnosis and surgical interventions.

Imaging is a vital tool in detecting and characterizing these malformations, providing the foundation for both prenatal and postnatal care. Advances in fetal ultrasonography (US) and magnetic resonance imaging (MRI) have significantly enhanced the ability to identify thoracic abnormalities during pregnancy [4,5]. This allows clinicians to anticipate potential management challenges at delivery and in the early stages of neonatal care, while also offering parents insights into the prognosis. Recognizing the distinct imaging features of various intrathoracic conditions in the fetus is critical for guiding appropriate care for both the mother and the infant.

Although prenatal imaging using US and MRI is effective for identifying lung abnormalities, these methods are not always sufficient to fully characterize the nature of the lesions. Additionally, lesions that seem to resolve in utero may still be visible after birth. Therefore, it is standard practice to perform a postnatal contrast-enhanced computed tomography (CT) scan before 6 weeks of age or between 3 and 12 months [5]. CT, often combined with CT angiography (CTA), is usually necessary for a definitive diagnosis and for planning surgical interventions. Multi-detector chest CT angiography is also important for evaluating potential blood supply from outside the lungs [5].

Despite the advancements in prenatal imaging, postnatal CT remains a crucial tool for accurately diagnosing CLMs and determining the most appropriate treatment plan.

This article explores the current approaches to imaging in pediatric patients suspected of having CLMs, providing an overview of the latest radiological findings and clinical characteristics.

## 2. Magnetic Resonance Imaging

Prenatal MR imaging is commonly employed following an abnormal fetal ultrasound screening. The core of fetal MR imaging relies on T2-weighted single-shot rapid acquisition sequences; in fact, usually, the fluid-filled fetal respiratory system and lungs exhibit a higher intensity on T2 images compared to the chest wall, and as the pregnancy advances, the amount of alveolar fluid produced rises, which further amplifies the T2 hyperintensity of the lungs [2].

Fetal MR imaging is particularly valuable for assessing large congenital lung malformations (CLMs), which may result in pulmonary hypoplasia and/or breathing difficulties. The assessment of residual lung volume has been demonstrated to have a direct correlation with patient prognosis, making it a crucial factor in predicting clinical outcomes [2].

On ultrasound, the fetal lungs generally appear homogeneous and slightly more echogenic than the liver, with their echogenicity increasing as the pregnancy progresses. The presence of cysts or focal areas of increased echogenicity in the lung parenchyma may suggest a mass. MR imaging, using respiratory-gated T2-weighted fast spin echo sequences, can distinguish between consolidation, cystic lesions filled with air or fluid, and hyperinflated lung tissue, which may contain fluid at birth and air later [5].

It is desirable to include the four-chamber view of the fetal heart in routine screening during the second and third trimesters for all fetuses; in this view, the heart occupies about 25–30% of the thoracic volume and is positioned in the left anterior quadrant, near the midline. The axis of the heart forms a 45° angle with the midline [4]. A shift in the cardiomediastinal position can often serve as an early indicator of a unilateral chest mass or diaphragmatic hernia.

Postnatally, MR imaging offers a radiation-free alternative to CT for evaluating both solid and vascular components of CLMs, thanks to its superior tissue contrast resolution. It can be used as a complementary or problem-solving tool, often in combination with chest CTA for a more comprehensive assessment of CLMs [2].

On MR imaging, the trachea, bronchi, and lungs display high T2 signal intensity relative to the chest wall muscles due to the significant fluid content in these structures. As the lungs mature, increased alveolar fluid production further elevates the lung signal intensity relative to the liver. MR imaging can also be used to measure normal lung volumes, which increase as gestation progresses.

Dedicated lung MRI is highly effective in detecting, localizing, and classifying congenital bronchopulmonary foregut anomalies, with excellent inter-reader agreement. Gadolinium-based contrast agents are indispensable for detecting lung lesions and delineating pathological vessels. Recent advancements in sequence acquisition, including center-out 3D k-space techniques with ultrashort (UTE) and zero echo times (ZTE), have improved pulmonary MRI [6]. These methods are less sensitive to patient movement, reducing the need for sedation, and their short echo times improve proton-density weighting, leading to better visualization of both hyperdense and hypodense lung tissue [6].

Innovations such as iMoCo (iterative motion-compensation reconstruction) UTE MRI offer sharper anatomical images and higher signal-to-noise ratios compared to other motion-correction methods [6]. Dynamic pulmonary MRI allows for the measurement of various respiratory metrics, such as tidal volume, airway collapse, and global ventilation, in neonates with lung disease. This technique enables the assessment of lung ventilation without ionizing radiation, making it a valuable tool for evaluating lung function, similar to chest CT but with the advantage of being non-invasive.

## 3. Computed Tomography

Chest CTA is the preferred imaging modality for the postnatal study of CLMs due to its capacity to thoroughly assess lung parenchyma and identify subtle vascular abnormalities [2]. To optimize the detection of abnormal vasculature, CTA should cover the area from the lower neck to the mid-abdomen [2]. Modern CT scanners, featuring advanced 320-row detector arrays, enable rapid imaging of the entire chest in infants in just a fraction of a second. These scanners are capable of capturing up to 16 cm along the z-axis (craniocaudal length) in one rotation [7].

Another cutting-edge technique, dual-source CT, uses two X-ray tubes and two detector arrays positioned at a 95° angle to each other. This configuration allows for the creation of two overlapping spiral datasets, scanning the same tissue volume in half the time of single-source scanners [7]. Together with faster tube rotation, larger detector arrays, and immobilization tools like vacuum splints to minimize patient movement, these advancements have transformed pediatric chest imaging. These advances have transitioned practice from requiring general anesthesia and breath-holding to ultrafast, high-quality scans with minimal motion artifacts, eliminating the need for sedation. Despite the elevated heart rates commonly seen in neonates, high-pitch CT, especially following the administration of intravenous contrast material, can deliver clear images of fast-moving structures such as pulmonary veins [7].

Sedation requirements for chest CTA are influenced by the patient’s age and their capacity to follow breathing instructions. However, the majority of chest CTA scans can be performed without sedation in infants under one year old, using multidetector CT in turbo flash spiral mode with a free-breathing approach.

In non-symptomatic children, it is best to delay imaging during the early neonatal period due to delayed resorption of fetal pulmonary fluid, which may limit lesion characterization. CTA imaging is most effective when performed after 4 weeks of age [2].

Despite its advantages, CT imaging has some limitations, including relatively high radiation doses and potential motion artifacts in children unable to cooperate with breath-holding. Technical parameters for CT imaging, such as tube current, kilovoltage, collimation, and table speed, may vary depending on the patient’s size and the CT scanner model. Generally, for patients under 10 kg, a current of 40 mA is used, whereas those over 50 kg receive 100–120 mA. Patients under 50 kg are imaged at 80 kVp, and larger patients require 100–120 kVp. Optimal imaging is achieved with multidetector CT, with collimation of 0.75 mm for 16-row CT, 0.625 mm for 32-row CT, and 0.6 mm for 64- and 96-row CT.

After obtaining the axial CT dataset, 3D reconstructions of the lung, airway, and blood vessels can be created, offering essential insights for assessing abnormal vessels and assisting in surgical planning.

## 4. Ultrasound

Ultrasound (US), due to its availability and the absence of radiation, is often the first approach in fetal and neonatal assessment of lung malformations. Its limits are represented by the poor panoramics and dependence on operator experience.

Lesions are assessed using orthogonal planes to obtain comprehensive imaging from multiple angles, and when feasible, high-frequency linear transducers (ranging from 12 to 15 MHz for postnatal children) are employed to achieve optimal resolution, allowing for precise visualization of the lesion’s characteristics [3]. On US, the fetal lungs typically appear homogeneous in texture, displaying a uniform consistency without any noticeable abnormalities; they are slightly hyperechoic, meaning they reflect more ultrasound waves and appear brighter than the adjacent liver parenchyma [4]. This subtle difference in echogenicity helps distinguish the lungs from nearby structures, such as the liver, during fetal imaging. Lesions associated with pulmonary malformations most commonly present as echogenic masses within the chest, but in rarer cases, they may appear in the abdomen, particularly in instances of extralobar pulmonary sequestrations [3]. Additional characteristics that may be observed with US include the presence of cystic formations, pleural effusions, or hydrops [3]. In cases involving large unilateral lesions, the mass effect can lead to a contralateral mediastinal shift, displacing the structures of the opposite side of the chest. On prenatal ultrasound, the presence of a cystic formation or a localized area of increased echogenicity within the lung parenchyma may indicate the potential presence of an underlying CLM [4]. Color-Doppler-ultrasound (CDUS) enhances the evaluation by providing insight into the associated vascular anatomy, aiding in the identification of abnormal blood vessels, such as those found in conditions like lung sequestration. These findings serve as important markers for further investigation and can aid in the early detection of such anomalies.

In children, the use of lung US is very limited. This is primarily due to the ossification of the bony thorax, which restricts the ability to obtain adequate sonographic windows. Additionally, as the lung volumes increase and air accumulates within the lungs, it obstructs the transmission of the ultrasound beam, further hindering effective imaging. These factors make it challenging to visualize and assess CLMs in older pediatric patients using ultrasound alone.

## 5. Malformations of the Pulmonary Parenchyma

### 5.1. Congenital Pulmonary Airways Malformations (CPAM)

**Figure 1:**
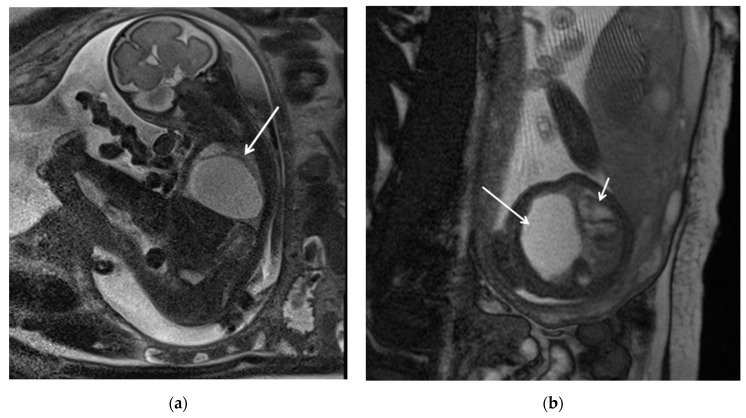
CPAM type I. HASTE oblique sagittal (**a**) and True FISP (**b**) MRI scans taken at 28 weeks of gestation reveal a large fluid-filled mass with bright T2 signal in the right chest (arrows). This abnormality results in a shift of the heart toward the left side of the mediastinum.

**Figure 2:**
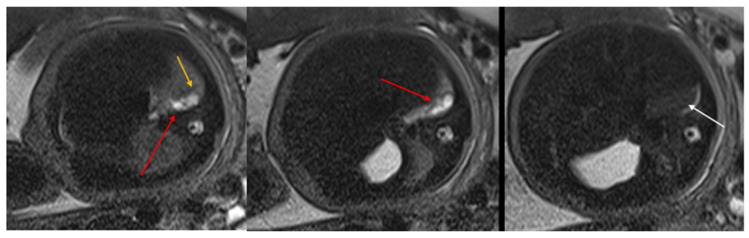
CPAM type II. Axial HASTE T2 MR images show a hyperintense lesion characterized by at least six cystic images (red arrow) of 2–6 mm at the lung base, with extension to the right posterior lung (orange arrow). No compression on the diaphragm or mediastinum. Minimal pleural effusion layer (white arrow).

**Figure 3:**
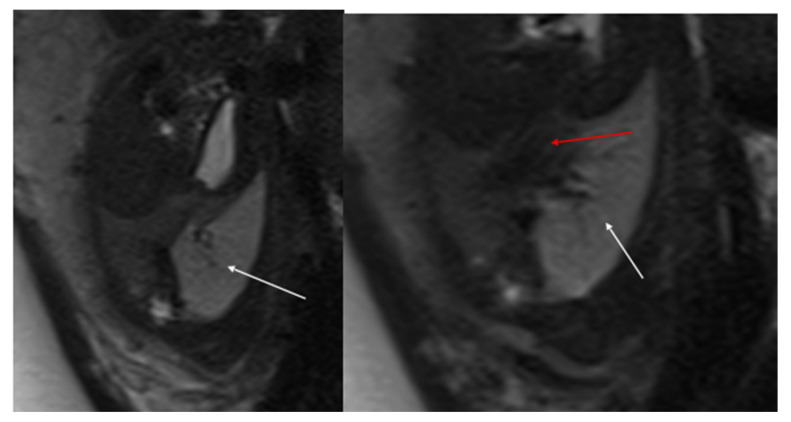
CPAM type III. Coronal T2 HASTE image shows, diffuse hyperintensity of the entire left lung (white arrow), without evidence of recognizable cystic structures. Note the normal intensity of the right lung (red arrow).

Congenital pulmonary airway malformations (CPAM) are subdivided into five types (0–4), with each type characterized by distinct features: Type 0 involves the trachea and mainstem bronchi and is lethal postnatally; Type I affects the bronchi and proximal bronchioles and is associated with large cysts (Figure 1); Type II involves the bronchiolar area with smaller cysts (less than 2 cm) (Figure 2); Type III affects the bronchiolar/alveolar duct region, often appearing as homogeneous hypodense masses on CT (Figure 3); and Type IV involves the alveolar/saccular region, causing unlined cysts [8].

Types I and II are typically visible on radiographs as lucencies, often associated with mediastinal shift, while CT imaging provides better characterization of cyst size, helps detect smaller cysts (type II), and identifies associated malformations (cardiac for type I, renal for type II) [8]. Imaging is less accurate in distinguishing type IV from type I due to their similarities [8]. The communication between the proximal airways and the vascular supply of CPAM can help differentiate it from pulmonary sequestration.

CPAMs are usually unilateral and may present as single or multiple cystic lesions; in neonates, the cysts may be fluid-filled, showing air-fluid levels or appearing solid [8]. CPAMs usually obtain their blood flow from the pulmonary artery and return it through the pulmonary veins, although hybrid lesions can have a systemic source of blood.

CTA with maximum intensity projection (MIP) reconstruction is the gold standard for postnatal evaluation of CPAM, offering the highest spatial resolution and sensitivity. The technique is recommended for identifying abnormal vasculature, often associated with CLMs, with anatomical coverage extending from the lower neck to the mid-abdomen to capture any abnormal vessels. CTA studies can be completed successfully without sedation; in fact, a recent study demonstrated that chest CTA performed without general anesthesia resulted in minimal motion artifacts. For accurate characterization, chest CTA post-processing techniques, such as multiplanar reformats and 3D reconstructions of the airway and vasculature, are essential. Studies have shown that although axial multidetector CT images can accurately diagnose CLM type, location, mass effect, and anomalous arteries, additional post-processing with multiplanar reformats and 3D reconstructions significantly enhances diagnostic value by improving detection of anomalous vessels, which is important for surgical planning.

**Figure 4:**
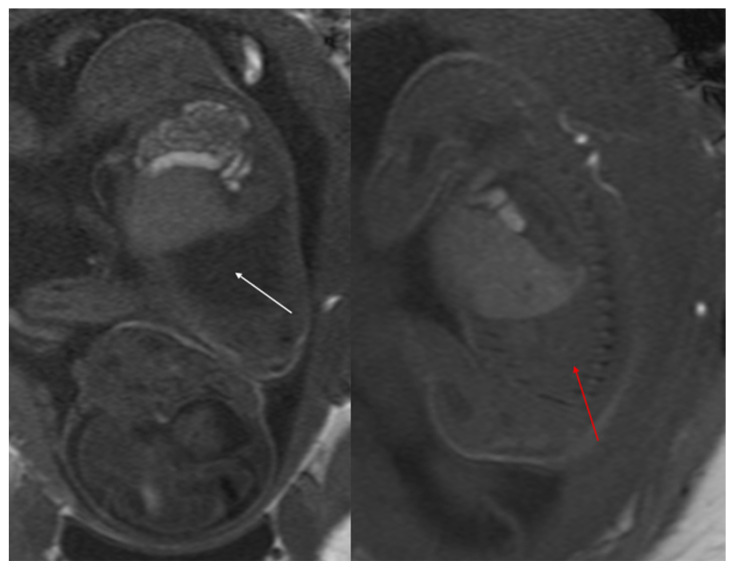
Sagittal T1w FLASH MRI sequence shows increased signal intensity in the left abnormal pulmonary parenchyma (white arrow) and the compressed signal of the right lung (red arrow). A differential diagnosis to consider is congenital lobar overinflation (CLO), where a more homogeneous signal intensity would typically be observed.

In asymptomatic children, it is advisable to avoid imaging in the early neonatal period due to the challenge of distinguishing delayed resorption of fetal pulmonary fluid from fluid-filled CPAM cysts. In cases of congenital lobar emphysema (CLO), hyperinflated lungs distal to the abnormal bronchus may remain fluid-filled, not yet showing typical hyperinflation (Figure 4).

**Figure 5:**
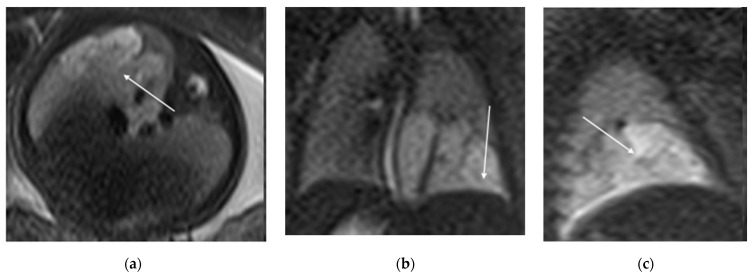
T2 HASTE axial plane (**a**), coronal (**b**) and sagittal (**c**): shows a multicystic brilliant formation (white arrow), at the level of the right and middle lower lobes. The lesion represents 55% of the right lung volume.

MRI is an alternative cross-sectional imaging modality, particularly with newer, faster sequences that improve detection of CLMs within aerated lungs, such as steady-state acquisition and single-shot fast spin echo (Figure 5). MRI can often be performed without sedation for infants under six months of age and older children who can follow breathing instructions. For children between these age groups, sedation may be necessary due to the length of the study. Chest MRI typically covers the lower neck to mid-abdomen, and acquisitions are performed in three planes, often with intravenous contrast. MR imaging also helps in surgical planning by accurately detecting soft tissue and fluid components within larger CLMs. Notably, all parenchymal CLMs demonstrate reduced enhancement at peak pulmonary perfusion after IV contrast administration.

In summary, both chest CTA and MRI are critical tools for evaluating congenital lung malformations, each offering unique advantages in terms of sensitivity, spatial resolution, and the ability to assess abnormal vasculature, which is vital for surgical planning.

### 5.2. Bronchopulmonary Dysplasia

Bronchopulmonary dysplasia (BPD) arises from a multifaceted process where various prenatal and/or postnatal factors disrupt the development of the lower respiratory system, resulting in a chronic, debilitating condition marked by ongoing lung dysfunction, recurrent respiratory issues, and a decline in both quality of life and life expectancy [9]. The lung vascular abnormalities that accompany BPD can lead to pulmonary arterial hypertension. Prematurity itself contributes to a heightened risk of long-term respiratory complications [9].

The use of CT early in BPD is most limited by the transportation of unstable infants to the CT scanner and, to a lesser extent, by concern about radiation [10]. However, the association of CT findings with clinical course, pulmonary function, and disease progression suggests that CT could play an increasing role in evaluating preterm babies that goes beyond problem-solving. A non-contrast chest CT performed at discharge from the NICU with a non-sedated feed-and-swaddle technique has the potential to provide information that would identify suspected findings, predict the early clinical course, and potentially decrease hospitalization in the first year after birth [10].

As a result of preterm birth, the central airway tends to be narrower, more flexible, and more vulnerable to injury, particularly in neonates who undergo endotracheal intubation and require positive pressure ventilation. Despite the significant role of central airway disease in BPD, our understanding remains limited, as the diagnosis usually depends on direct examination through bronchoscopy. However, recent advancements in CT and MRI offer a noninvasive approach to assess the neonatal airway without the need for sedation. MRI, in particular, provides the advantage of avoiding ionizing radiation, making it a promising tool for longitudinal studies on central airway disease in BPD.

### 5.3. Bronchopulmonary Sequestration (BPS)

Pulmonary sequestration, the second most common lung lesion after CPAM, is characterized by a portion of lung tissue that does not connect to the tracheobronchial tree and is instead supplied by a systemic artery, usually originating from the thoracic or abdominal aorta [3,11]. It can be classified into two types: intra-lobar and extra-lobar sequestration (Table 1).

Extra-lobar sequestration is characterized by a separate visceral pleura, with venous drainage into the systemic veins [11]. This form is most commonly diagnosed prenatally. CT angiography with 3D reconstruction is highly informative in identifying anomalous feeding arteries and drainage veins, which is crucial for distinguishing intra-lobar from extra-lobar sequestration [12].

Intra-lobar sequestration, on the other hand, is typically located within the normal lung parenchyma, often in the posterobasal segment of the left lower lobe. It is characterized by an anomalous artery arising from the abdominal aorta and a vein draining into the pulmonary venous system [11]. The CT appearance of sequestration can range from a homogeneous soft tissue mass to a cystic lesion containing air or fluid, with hybrid forms also possible.

**Figure 6:**
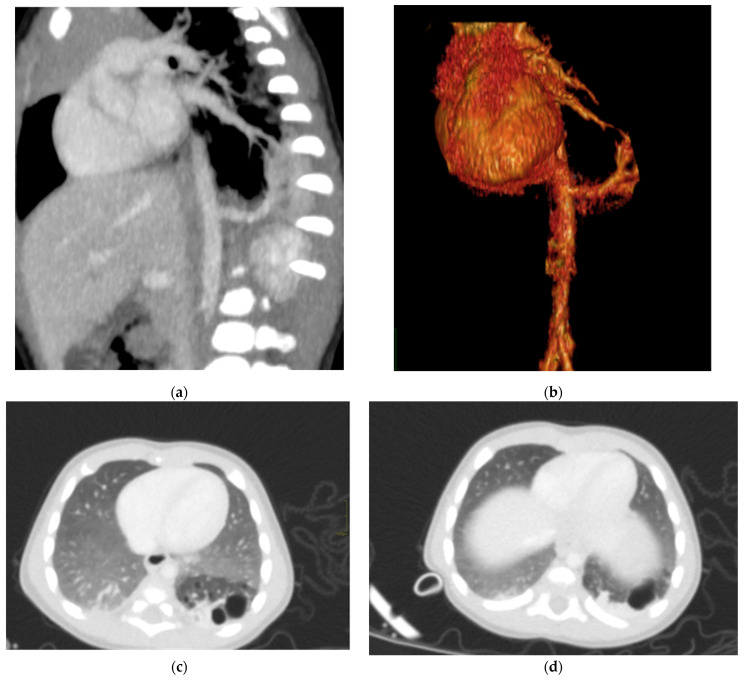
Hybrid congenital malformation consisting of lung seizure and CCAM. CECT MPR reconstruction (**a**), VR (**b**), and axial (**c**,**d**) images show the presence of a lobulated-margin formation located in the lower lobe of the left lung, which is consists of multiple cystic formations (diameters ranging between a few millimeters and 1.5 cm) and a consolidation zone corresponding the costodiaframmatic recess, which is supplied by a voluminous arterial branch originating from the left lateral wall of the aorta in the thoraco-thoracic passage.

In terms of location, about 85–90% of sequestrations are supradiaphragmatic, with the remaining 10–15% being subdiaphragmatic [11]. Approximately 90% of sequestrations are located on the left side. The systemic arterial supply typically comes from both the lower thoracic and upper abdominal aorta (Figure 6).

**Figure 7:**
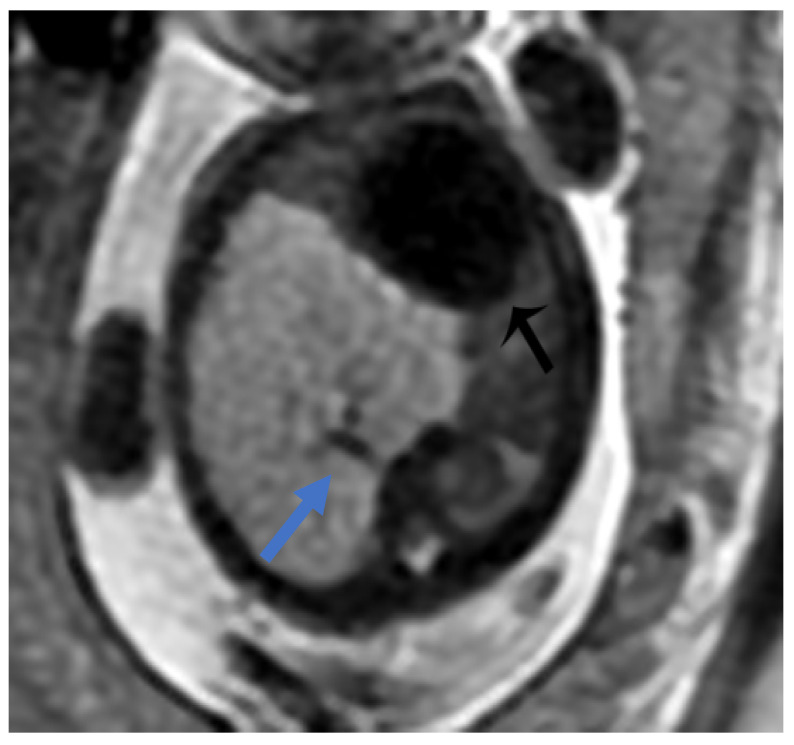
BPS at 27 weeks gestation. The coronal image shows a mass with higher signal intensity than the normal lung but lower signal intensity than amniotic fluid. The consolidation shifts the heart to the right (black arrow). There is a feeding artery from the aorta to suggest the diagnosis of sequestration (blue arrow).

In prenatal diagnosis, MRI is valuable for better delineating and locating the mass. It also helps in assessing the contralateral lung. Key diagnostic signs on MRI include higher signal intensity than normal lung tissue, lower signal intensity than amniotic fluid, and the visible supply artery arising from the aorta (Figure 7) [8,11].

**Figure 8:**
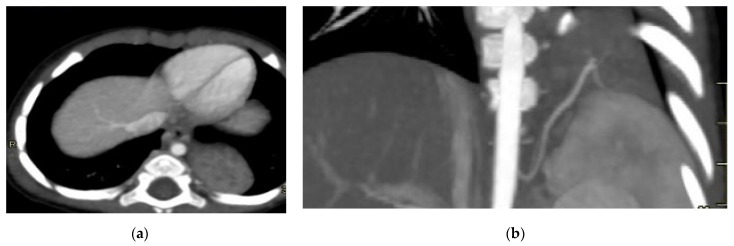

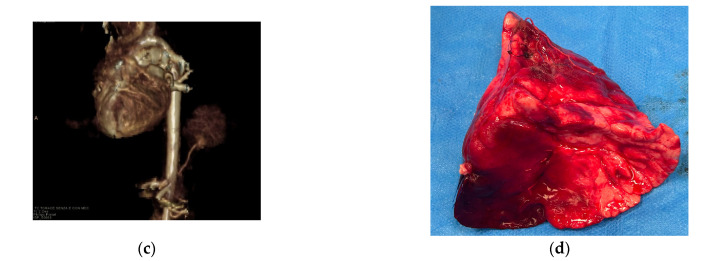
Intra-lobar bronchopulmonary sequestration. The Contrast-enhanced computed tomography (CECT) axial (**a**), coronal MPR reconstruction (**b**), and volume rendering reconstruction (**c**) images show a parenchymal consolidation located in the lower left lobe supplied by an afferent artery originating from the suprarenal tract of the abdominal aorta. Intraoperative findings confirmed the bronchopulmonary sequestration (**d**).

For postnatal evaluation, CT and CT angiography are the methods of choice, as they provide clear visualization of vascularized parenchymal consolidation with arterial supply from the descending or abdominal aorta (Figure 8).

### 5.4. Pulmonary Hypoplasia and Aplasia

Arrest in lung development causes pulmonary hypoplasia, pulmonary aplasia or pulmonary agenesis, depending on the time of the arrest and the resulting amount of absent parenchyma [11].

Pulmonary agenesis is the complete absence of bronchi, pulmonary vessels, and pulmonary parenchyma. Left lung agenesis is the most common, observed in 70% of cases [11].

Pulmonary hypoplasia can be primary or secondary; the primary form is usually fatal, although milder forms are known to be associated with Down syndrome [11].

Most cases of pulmonary hypoplasia are seen secondary to oligohydramnions, lung masses, or congenital diaphragmatic hernia, which cause a reduction in the space inside the chest or alterations in fluid dynamics.

In the fetus, MRI is useful for assessing thoracic masses that can cause hypoplasia and to evaluate lung volumes due to excellent soft tissue contrast. It is also a useful diagnostic aid in the presence of oligohydroaminos, which limits the diagnostic reliability of ultrasound [11].

The diagnosis of pulmonary hypoplasia is based on the measurement of the total volume of the lungs as well as on the evaluation of the intensity of the lung signal.

MRI shows the hypointense T2 signal of the hypoplastic lung compared to the normal appearance of the lung, while the progressive hyperintensity typically observed during pregnancy is not appreciated [11].

CT is very rarely used in the study of pulmonary hypoplasia. It is more commonly used in the evaluation of pulmonary aplasia, showing the absence of the lung and the ipsilateral pulmonary artery, which accompanies mediastinal shift towards the pathological side and the compensatory hyperinflation of the contralateral lung [8].

### 5.5. Congenital Lobar Overinflation (CLO)

Also known as congenital lobar emphysema (CLE), CLO is a rare congenital malformation characterized by overinflation of the lung. It may cause respiratory distress in newborns. Its incidence is 1/20,000–30,000 live births [13].

It is usually unilateral and generally affects one lobe; however, the involvement of more pulmonary lobes and bilateral lesions has been described. The most common observation of this disease is left upper lobe involvement (43%), followed by right middle lobe (32%) and right upper lobe (21%) involvement. Congenital cardiac defects are likely to accompany CLO [11,13].

In the prenatal period, prenatal USG shows hyperechogenicity in lung segments without abnormal blood flow; a mediastinal shift and/or polyhydramnios may accompany this situation [13]. When an echogenic lung is identified on ultrasound, differential diagnoses to consider include CLE, congenital cystic adenomatoid malformation, and pulmonary sequestration [13].

At fetal MRI, CLO is characterized as an area of increased T2 signal with a lobar distribution; it is usually less hyperintense when compared to BPS and the lung structure generally appears intact with stretched hilar vessels [11].

After birth, CT is the gold standard for studying the anatomy and features of the affected lobe, evaluating the status of the adjacent lobes (compression or atelectasia), and determining whether the contralateral lung tissue is hypoplastic [8,13].

## 6. Congenital Tracheobronchial Branching Anomalies

Most of these anomalies involve displaced (abnormal bronchus origin, where the normal bronchus supplying the corresponding parenchyma is absent) or supernumerary bronchi (a supernumerary bronchus exists alongside the normal bronchus) [14]. The supernumerary bronchus may terminate blindly in the parenchyma of the corresponding normal bronchus or be associated with ventilated tissue [14].

### 6.1. Tracheal Bronchus

A tracheal bronchus, also known as “pig bronchus”, is defined as a right ULB originating from the trachea, generally 2–6 cm proximal to the carina [8,14].

A tracheal bronchus is almost invariably located on the right side, and it is seen in 1.5–2% of the pediatric population [14]. In children, it may be associated with recurrent pneumonia and other respiratory diseases.

CT images generally show that the tracheal bronchus originates from the distal trachea, typically 2–6 cm above the carina, but the distance may be lesser than 2 cm [8,14]. It is very important to report the distance between the carina and the tracheal bronchus to the anesthesiologist. A right-sided tracheal bronchus can provide ventilation to the entire right upper lobe or as limited as a subsubsegment [14].

The tracheal bronchus is classified into displaced or supernumerary types, based on whether the normal branching pattern of the right mainstem bronchus is present [15]. Displaced types are more frequent than the supernumerary bronchi.

### 6.2. Accessory Cardiac Bronchus (ACB)

ACB is the only bronchus originating from the medial wall of either the RMB or the intermediate bronchus, but it can also occasionally occur on the left side [14]. Patients may experience recurrent cough, pneumonia, or dyspnea; rarely, lung tumors can arise from ACB [14]. On chest CT, the abnormal bronchus often arises from the medial aspect of the proximal third of the intermediate bronchus. The surrounding lung tissue may appear as a mass at the bronchial tip or as a ventilated lobule, which is typically delineated by an accessory fissure [14].

### 6.3. Bridging Bronchus

A bridging bronchus is an extremely rare aberrant bronchus that partially or totally supplies the right lung but that originates from the LMB [14]. Bridging bronchi can be divided into two main subtypes.

**Figure 9:**
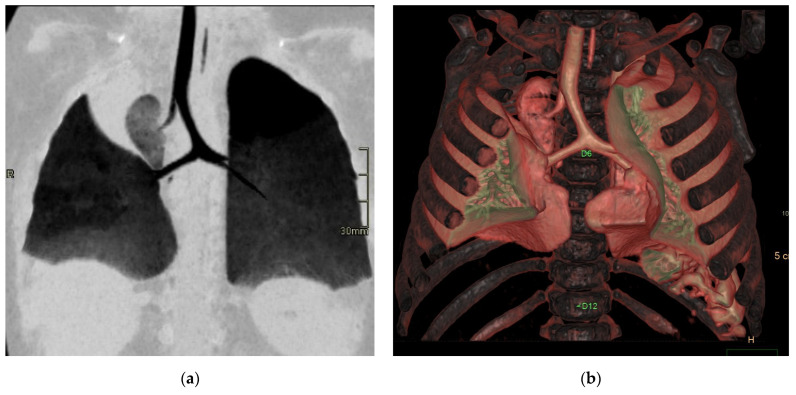
Bridging bronchus type 2. Non-contrast CT coronal (**a**) and VR (**b**) images reveal the RMB ending in a diverticulum. The right lung is ventilated by a displaced RMB arising from the LMB, forming a pseudo-carina, that is at the T6–T7 level and has an inverted T appearance.

In type 1, the right main bronchus (RMB) terminates in the right upper lobe (ULB), while the intermediate bronchus abnormally arises from the left main bronchus (LMB), creating a pseudocarina; either the right bronchus or a bridging bronchus generally supplies the middle lobe [14]. Type 2 is often associated with right lung hypoplasia (Figure 9). In this type, the RMB may be absent or end in a diverticulum. The entire right lung is ventilated by a displaced RMB originating from the LMB, forming a pseudocarina [14].

In this condition, the carina faces thoracic vertebrae T4 and T5; the pseudocarina, instead, is at the T6–T7 level and has an inverted T appearance [14].

### 6.4. Situs Anomalies

Situs solitus corresponds to the usual arrangement of the organs, which means that the right ULB is in an eparterial position, and the left ULB is in a hyparterial position; situs inversus consists of the mirror images of situs solitus which means that the left and right ULB are in the opposite positions [14]. The term situs ambiguus includes other arrangements; in particular, right isomerism (also known as asplenia syndrome) is characterized by both ULBs in eparterial positions. On the contrary, left isomerism (also known as polysplenia syndrome of Ivemark syndrome) is defined in the of bilateral hyparterial bronchi [14].

On CT, the most reliable indicator of bronchial situs is the relationship between the ULB and the ipsilateral pulmonary artery [14].

### 6.5. Congenital High Airway Obstruction Syndrome (CHAOS)

Congenital high airway obstruction syndrome (CHAOS) is an exceptionally rare and typically fatal condition resulting from complete or partial congenital obstruction of the upper airways [16,17].

Most lesions associated with CHAOS result from upper airway obstruction. Notably, pulmonary hypertension develops rapidly due to fluid accumulation in the lungs, leading to compression and displacement of the heart, which is also abnormally small in size [16]. In patients with laryngeal or tracheal atresia, the fluid is not drained into the amniotic cavity but it remains trapped within the lungs, causing the hyperechogenic appearance on ultrasound and the grossly enlarged lungs. Consequently, the heart is typically displaced, small and/or compressed; the risk of cardiac failure and ascites rises because of the high intrathoracic pressure [18].

Fetal MRI can aid in making an accurate diagnosis, determining the level of obstruction, and assessing which patients are suitable for in utero surgery. Of course, the effectiveness of MRI investigation depends on the gestational age.

MRI demonstrates increased lung volumes with diffuse increased T2 signal due to higher fluid content; the large lungs cause flattening or inversion of the diaphragm, anterior displacement of a small heart, and hydrops [11]. Dilatation of the airway to the level of the obstruction is present and is important for surgical planning. Hydrops is a sign of poor prognosis [11] MRI is effective in precisely localizing the level of obstruction, helping to determine whether fetal intervention or neonatal management through the ex utero intrapartum treatment (EXIT) procedure is more appropriate [4]. It is very sensitive in identifying potential respiratory tract compression that could cause life-threatening airway obstruction at birth, enabling optimal pre-labor planning for the EXIT procedure [4].

## 7. Conclusions

Congenital lung malformations are amongst the most common causes of thoracic surgery in infancy because of possible infective and respiratory consequences. The diagnosis can be established antenatally using US and MRI [18,19].

Postnatally, TC and MRI are both accurate modalities for the diagnosis; the choice between these two options depends on the radiologist’s expertise and scanner availability. CT is better for the assessment of parenchymal findings and any associated vascular abnormalities, which are well visualized with CTA postprocessing techniques such as multiplanar reformats and 3D/4D reconstructions. Multicenter studies are needed to assess the clinical role of new ultrashort echo time (UTE) MRI sequences for evaluating lung parenchyma, and dynamic MRI for evaluating lung perfusion, respectively.

In conclusion, advanced imaging modalities provide an accurate pre- and neonatal evaluation in pediatric patients with congenital lung malformations, which is useful for choosing the correct management and therapy.

## Figures and Tables

**Table 1:** Different features of intra-lobar and extra-lobar sequestration.

	Intra-Lobar Type	Extra-Lobar Type
**Incidence**	75%	25%
**Cause**	Acquired or congenital	Congenital
**Timing of Diagnosis**	Childhood or adulthood	Prenatal–neonatal period
**Pleural Investment**	Within lobe without its own pleura	With its own lung pleura
**Arterial Supply**	Thoracic or abdominal aorta	Abdominal aorta
**Venous Drainage**	Pulmonary venous system	Systemic venous system
**Association with Other Congenital Anomalies**	Rare	Common

## Data Availability

No new data were created for this article.
